# Sex ratios in vocal ensembles affect perceptions of threat and belonging

**DOI:** 10.1038/s41598-024-65535-x

**Published:** 2024-06-25

**Authors:** Kelsey L. Neuenswander, Brianna M. Goodale, Gregory A. Bryant, Kerri L. Johnson

**Affiliations:** 1 Department of Communication, University of California, Los Angeles, 2225 Rolfe Hall, Los Angeles, CA 90095 USA; 2 Julius Clinical, Zeist, The Netherlands; 3 Department of Psychology, University of California, Los Angeles, USA

**Keywords:** Ensemble coding, Group perception, Auditory perception, Social cognition, Psychology, Human behaviour

## Abstract

People often interact with groups (i.e., ensembles) during social interactions. Given that group-level information is important in navigating social environments, we expect perceptual sensitivity to aspects of groups that are relevant for personal threat as well as social belonging. Most ensemble perception research has focused on visual ensembles, with little research looking at auditory or vocal ensembles. Across four studies, we present evidence that (i) perceivers accurately extract the sex composition of a group from voices alone, (ii) judgments of threat increase concomitantly with the number of men, and (iii) listeners’ sense of belonging depends on the number of same-sex others in the group. This work advances our understanding of social cognition, interpersonal communication, and ensemble coding to include auditory information, and reveals people’s ability to extract relevant social information from brief exposures to vocalizing groups.

## Introduction

Social cognition research has recently expanded to investigate *people* perception in addition to *person* perception^1,2^—groups of people have perceptible qualities that are separable from the characteristics of the individuals that make them up. Much of human social interaction and communication involves groups of people. For example, full-time college students often spend up to 20 h per week in a classroom. The average U.S. citizen spends over 50,000 h with coworkers throughout their lifetime^3^. Many individuals are involved in teams, clubs, and other social groups. Outside of formal group interactions, people perceive groups of strangers at restaurants, live shows, and sporting events. Thus, it is important to understand how groups (i.e., ensembles) are perceived.

Most research investigating ensemble perception has focused on the visual system’s ability to extract summary statistical information from a group, often in a brief glance^4^. Perceivers can accurately extract summary statistics of ensembles from low-level features such as hue^5,6^, brightness^7^, orientation^8–10^, spatial position^11–13^, and motion^14,15^, as well as mid-level features such as size^16,17^. Perceivers can also extract high-level features from ensembles including emotion^18^, family resemblance^19–22^, walk motion^23^, eye-gaze direction^24^, and social category membership^25–28^. Importantly, the social category membership of a group influences evaluations. Groups with more men are perceived as more threatening^25^ and more likely to harbor sexist norms^26^, whereas perceivers feel greater belonging in groups with more same-sex others^26^. Threat and belonging are particularly important social judgments for marginalized group members and can have critical impacts on welfare and safety. These judgments also influence adaptive behavior. When a group is perceived as threatening, individuals often avoid it^29–31^. When individuals feel that they fit or belong within a group, they tend to approach it^32–34^. Overall, observers efficiently get the gist of groups from their visual properties which has implications for group evaluations and subsequent social action.

Auditory or vocal ensembles are relatively understudied compared to visual ensembles. However, vocal ensembles are an important component of overall ensemble perception and present an adaptive problem for many species, including humans. Most generally, visual information is not always available. For example, in many environments, especially at night, groups can be heard but not seen, requiring the ability to extract information about them from only voices and other sounds. We should expect people to be attuned to auditory ensembles and be able to draw rapid inferences regarding socially relevant features such as whether the groups constitute possible adversaries or allies. Moreover, when visual information is available, it is sometimes obscured. In large group scenarios such as in the examples provide earlier (i.e., restaurants, live shows, and sporting events), we often hear group members before we are close enough to see them. In academic and professional settings, the use of telework on platforms such as Zoom, Microsoft Teams, and Webex has grown drastically in response to the Covid-19 pandemic^35,36^. Visual information is not always shared on these platforms—to combat “Zoom fatigue,” researchers are suggesting people keep their cameras off^37^. Overall, individuals must rely on auditory information when visual information is nonexistent or suboptimal, so we should expect an evolved auditory perceptual sensitivity to group-level information.

Despite vocal ensembles being relatively understudied, ensemble perception is expected to operate similarly across sensory modalities. Listeners can extract summary statistics of a group from low-level auditory features such as pure tones^38^ and sound textures^39^, as well as high-level features such as social category membership. For instance, perceivers use sexually dimorphic aspects of the voice (e.g., pitch and timbre) to accurately judge the ratio of men to women in a group after listening to very brief (1500 ms) audio recordings of 5 or 10 simultaneous voices^40,41^. However, no research has investigated whether summary statistics of auditory information have downstream consequences for social judgments of threat or belonging. Given that sex composition information affects social evaluative judgments in visual ensembles, and people can extract sex ratios from auditory ensembles, we should expect an effect of sex composition in vocal ensembles on social evaluations of vocalizing groups. The following studies address this by testing if listeners can extract the sex composition of a group from voices alone (Studies 1–3) and whether this influences perceivers’ feelings of threat (Study 2) and belonging (Study 3) toward the group.

In this paper, we differentiate between sex and gender. We refer to voice sex to reflect sexual dimorphism in voice properties, including fundamental frequency (*f*_o_; the acoustic correlate of perceptual pitch) and formants. However, because all voice recordings are from cisgender individuals, we also use the terms “men” and “women” to discuss group composition. For masculinity and femininity judgments, we refer to gender due to the full spectrum this encompasses. Participants only provided their gender identity and thus we refer to participant gender rather than sex.

## Study 1a

The primary aim of Study 1a was to demonstrate that listeners can accurately extract the sex ratio of a group from voices alone. This study extends the existing research investigating the perception of vocal sex ratios in five distinct ways^40,41^. First, vocal ensembles that consisted of twelve people rather than five or ten people were used to test whether listeners accurately extract summary statistics from larger groups. Second, sequential rather than simultaneous recordings of ensembles were used to emulate turn-taking scenarios in academic or professional settings (e.g., classroom, boardroom, panel). The decision to use sequential stimuli aligns with previous studies investigating auditory ensemble coding^38,42,43^. Third, the duration of our ensembles differed due to our sequential approach. While the overall length is longer than previously tested vocal ensembles (3000 ms vs. 1500 ms), the length of each individual voice recording within the ensemble is shorter (250 ms vs 1500 ms). In the visual literature, observers accurately extracted the sex ratio of a group after a mere glimpse^25,26^. Similarly, we tested if people could extract summary statistics of a group after hearing a brief snippet of their voices. Fourth, semantic content was controlled by using audio samples that used the same utterance (“hi”). Fifth, an additional dependent measure was collected to determine if accuracy estimates are cross modal; that is, whether the sex ratio of the group presented auditorily influenced the estimated visual appearance of the average group member. It is possible that low-level perceptual features of the voice activate mental representations of social categories that transcend sensory domains.

Participants listened to 50 vocal ensembles that varied in the ratio of men to women (0:12, 3:9, 6:6, 9:3, 12:0) and estimated the sex ratio and average facial appearance of the group. We predicted that our results would replicate and extend findings reported previously^40,41^. Specifically, we made the following predictions: (i) as the actual ratio of men to women in a group increases, the perceived ratio of men to women in a group will also increase, and (ii) as the actual ratio of men to women in a group increases, the perceived average group member will become more facially masculine.

### Method

All studies were approved by the UCLA Institutional Review Board and were performed in accordance with relevant guidelines and regulations. Data and analysis code are publicly available on OSF (https://osf.io/28gvd/).

#### Participants

An a priori power analysis was run to determine the recommended sample size for a CNC within-subjects linear effects mixed model with actual sex ratio of the group as a fixed effect and participants and targets as random intercepts. The letters N and C indicate whether the random factors in each pair are nested or crossed, respectively^44^. The first letter indicates that participants are crossed with actual sex ratio, the second letter indicates that targets are nested within actual sex ratio, and the third letter indicates that participants and targets are themselves crossed.

A priori power analyses for linear mixed models are complex given they require knowing numerous parameters such as the number of level-one groups, the estimated effect size, variance of random effects, covariance of random effects, regression coefficients, and the design effect^45–49^. To address these complexities, we were conservative in our sample size estimates. Our analysis determined that a sample size of 64 participants was needed to detect an effect size of 0.50 with 80% power and an alpha level of 0.05^46^. We exceeded this target number and recruited 96 individuals (Gender: 59% women, 39% men, 2% genderqueer; Race: 72% White, 12% Black, 9% Asian, 7% biracial/other; Age: *M* = 38.77 years, *SD* = 14.63 years, min = 19, max = 71) through an online participant pool, Prolific (https://www.prolific.co/), who were paid $3.00 for their participation in a 20-min study. We initially included participant gender as a potential moderator in all studies. However, participant gender did not significantly moderate any results in Studies 1 and 2 and was therefore dropped from the analyses.

#### Stimuli

Audio clips were sampled from voice recordings used in prior research (e.g.^50^), and also unpublished work. The voice recordings consisted of 104 individuals (52 cisgender men, 52 cisgender women) saying “Hi, I’m a student at UCLA”. Voices were recorded in a quiet room on a digital (16 bit, 44.1 kHz) recorder (Marantz PMD-660 or M-Audio MicroTrack 24/96) with a cardioid condenser microphone (AKG C535 EB) 15–20 cm away from the mouth.

Audio clips were trimmed to 250 ms in length and only included the word “hi.” A custom Python script was created to generate ensembles, such that voices were randomly selected within trial, but with replacement between trials, from the available bank of voice stimuli. Ensembles consisted of twelve voices that played sequentially. The order of the voices was randomized within each ensemble and varied in the ratio of men to women (0:12, 3:9, 6:6, 9:3, 12:0). A set of 10 unique ensembles was generated for each of the 5 possible sex ratios, resulting in a total of 50 ensembles that were presented to each participant.

#### Procedure

**Figure 1:**
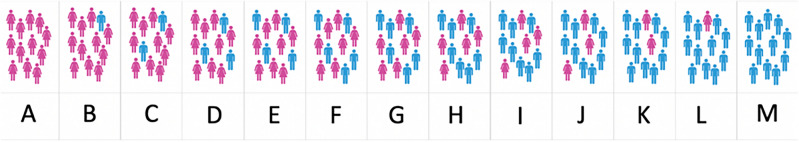
Sex ratio scale. Note: Which picture (labeled by letter) best represents the ratio of men to women you just heard?

**Figure 2:**
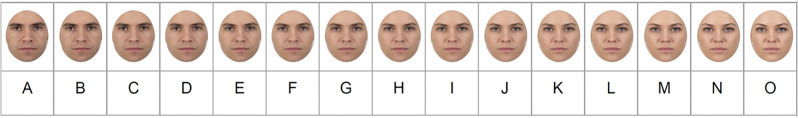
Gender morph scale. Note: Which picture (labeled by letter) best represents the average group member?

After completing an informed consent form, participants verified that they were wearing headphones and in a quiet environment. The study protocol consisted of two blocks presented in counterbalanced order. Participants completed 50 trials in each block, for a total of 100 trials. On each trial, participants listened to an ensemble and provided judgments. In one block, participants were asked to estimate the ratio of men to women in the ensemble using a stick figure scale (Fig. 1). In the other block, participants were asked to estimate what the average group member looked like on a gender morph continuum from very masculine to very feminine (Fig. 2). After completing these two blocks, participants provided us with demographic information and were debriefed.

### Results

The R packages “lme4” and “lmerTest” were used to create hierarchical linear models that accounted for within-subject variation and nesting within participants^51,52^. For all studies, we created models using the CNC design described above^44^ with actual sex ratio as a fixed effect and participants and targets as random intercepts. Significance was determined using traditional cutoff values of $\alpha$ = 0.05, and we conducted thorough checks for assumptions in our models, including normality assessments, to ensure there were no severe violations.

First, we tested whether participants were accurate in their perceptions of ensembles by regressing the estimated number of men in the group onto the actual sex ratio. Indeed, as the ratio of men to women increased, so did participants’ numeric estimates of men in an ensemble, *B* = 0.81, *SE* = 0.04, *t*(129) = 20.54, *p* < 0.001. Next, we tested whether the actual number of men in each ensemble affected how masculine or feminine the average group member was perceived to be. As the number of men in the group increased, participants estimated that the average group member was more facially masculine, *B* = 0.88, *SE* = 0.03, *t*(108) = 33.20, *p* < 0.001.

### Discussion

The results of Study 1 supported both of our hypotheses. As the actual ratio of men to women in a group increased, so did participants’ estimated sex ratio. Furthermore, as the actual number of men to women in a group increased, so did the perceived facial masculinity of the group. In sum, listeners were calibrated to the group’s sex ratio after briefly hearing their voices and this had cross-modal influence on predicted group appearance.

Given the sequential nature of our stimuli, it is possible that extracting the sex ratio from the group was the result of participants counting the number of men and women in the group. This is unlikely given the short duration of voices within the presented ensemble (250 ms). Nonetheless, to address this concern, we ran an additional study in which participants estimated the sex ratio while simultaneously undergoing a cognitive load.

## Study 1b

Study 1b replicated the accuracy block of Study 1a. However, participants were told that the study was designed to test the influence of auditory information on their memory for different shapes. Instead of listening solely to vocal ensembles, participants were tasked with memorizing the position of four different shapes on their computer screen while listening to a vocal ensemble. If extracting the sex ratio of a vocal ensemble is an efficient process, as we expect, then participants should be able to accurately estimate the sex ratio of a group even with the presence of a cognitive load.

### Method

#### Participants

130 individuals were recruited through Prolific (https://www.prolific.co/) and participated in this 10-min study in exchange for $1.50. Three individuals were excluded from analyses for providing identical judgments across all trials, and 14 individuals were excluded for reporting audio issues. This yielded a final sample size of 113 (Gender: 52% women, 47% men, 1% genderqueer; Race: 67% White, 18% Black, 6% Asian, 9% biracial/other; Age: *M* = 40.8 years, *SD* = 15.0, min = 19, max = 79).

#### Ensemble stimuli

The ensemble stimuli were the same stimuli used in Study 1a.

#### Shape stimuli

We selected four shapes to use in the cognitive load memory task: square, circle, triangle, and diamond. Each shape was outlined in black, had a transparent center, and measured 1 inch in height.

#### Procedure

After completing an informed consent form, participants verified that they were wearing headphones and in a quiet environment. Participants were instructed that the study was investigating the effect of audio information on their memory for shapes. The instructions specified that they would hear a group of voices while they were briefly presented with four shapes simultaneously in varying corners on the screen (top left, top right, bottom left, bottom right). Participants were told that it was important for them to try to remember the various positions of the shapes on the screen for a subsequent memory test.

On each trial, a randomly selected ensemble was played that varied in sex ratio ranging from all men to all women (0:12, 3:9, 6:6, 9:3, 12:0). While the ensemble played, the location of the four shapes was randomly shown on the screen (top left, top right, bottom left, bottom right). Immediately after hearing the vocal ensemble, participants were asked “where was the [circle/square/diamond/triangle] located on the screen?” followed by four multiple choice options: top left, top right, bottom left, bottom right. After they provided their answer, they were then asked to estimate the ratio of men to women in the ensemble using the same visual scale from Study 1a (Fig. 1). The study protocol consisted of 50 total trials. At the end of the study, participants provided demographic information and were debriefed.

### Results

First, we tested whether participants were accurate in efficiently extracting the sex ratio of a group by regressing the estimated number of men in the group onto the actual sex ratio. As the ratio of men to women increased, so did participants’ numeric estimates of men in an ensemble, *B* = 0.75, *SE* = 0.03, *t*(138) = 24.48, *p* < 0.001. The effect size was slightly smaller than Study 1a (0.75 versus 0.81, respectively) which was unsurprising given the addition of a cognitive load (Table 1). However, participants were still notably efficient and accurate at estimating the sex ratios of groups even when that was not their primary goal. This result suggests that extracting information about a group’s sex ratio from their voices is a relatively effortless process, like extracting sex ratio information from visual ensembles^25,26^.

**Table 1:** Comparison of statistical effects for Studies 1–3.

	Perceived sex ratio				Perceived facial gender			
	*B*	*SE*	*t*	*p*	*B*	*SE*	*t*	*p*
Study 1a	0.81	0.04	20.54	< 0.001	0.88	0.03	33.20	< 0.001
Study 1b	0.75	0.03	24.48	< 0.001	–	–	–	–
Study 2	0.84	0.04	23.03	< 0.001	0.85	0.03	32.50	< 0.001
Study 3	0.80	0.04	19.93	< 0.001	0.88	0.03	33.80	< 0.001

Effects of actual sex ratio on perceived sex ratio and perceived facial gender.

### Discussion

The results from Study 1b replicated the findings from Study 1a with a cognitive load. Even when participants’ primary motivation was to memorize basic shapes, they were still attuned to the relative ratio of men and women within a group, suggesting that extracting group sex information can be accomplished efficiently and with little awareness. In fact, although the effect size was smaller in Study 1b with the cognitive load, it was not drastically different from Study 1a. Because of this, the cognitive load task was dropped from future studies.

Thus far, Studies 1a and 1b established that listeners rapidly and accurately represent the relative sex ratio of an ensemble. However, these studies did not test downstream consequences of accuracy on judgments of the group. Evidence suggests that perceivers draw evaluative inferences about a group based on its sex composition. For instance, male-dominated groups are judged as more threatening^25^. Multiple factors account for this. First, men have historically held historic social, economic, and political power imbalances. According to social identity^53–55^ and intergroup threat^56,57^ theories, groups with more power are often seen as more threatening, especially by lower-power groups. Second, men are more likely than women to pose a threat, as reflected in violent crime statistics worldwide^58,59^. Third, physically strong men are more militant^60^ and masculine voices are a reliable indicator of men’s physical strength (i.e., threat potential)^61^. Thus, it is evolutionarily adaptive to ascertain the number of men in a group quickly and accurately. However, accurate detection of a group’s composition may inadvertently undermine the accuracy of threat detection when observers use these percepts as a proxy for threat. Specifically, perceptions of a group’s sex composition might elevate or mitigate perceived threat when men are common or rare, respectively.

Another important social judgment that might be influenced by the sex ratio of vocal ensembles is a sense of fit or belonging. Humans are fundamentally motivated to achieve a sense of belonging^62^. In the visual literature, social belonging depends on the number of same-gender others in the group^26^. That is, men feel more belonging in groups with more men, and women feel more belonging in groups of more women. Given that voices communicate information that perceivers are attuned to, and perceivers are motivated to determine whether they fit within a group, it is likely that perceptions of belonging are tethered to the number of similar others (i.e., same-gender) in the group.

To test the influence of auditory ensemble coding on evaluative judgments, the following studies assessed whether the sex composition of a group influenced perceptions of threat (Study 2) and belonging (Study 3).

## Study 2

Study 2 aimed to replicate and extend the findings from Study 1 with a specific focus on understanding whether group sex composition influenced evaluative judgments of threat. Participants listened to ensembles and estimated the sex ratio of the group, the perceived average facial appearance of the group, and the perceived threat of the group. Given that threat judgments appear heavily tethered to the number of men within an ensemble^25^, we predicted that as the actual ratio of men to women in a group increases, perceived threat will also increase.

### Method

#### Participants

Ninety-two participants from Prolific completed this 30-min study in exchange for $4.50. Twenty-one participants were excluded from analyses due to identical responses on every scale item. This yielded a final sample of 71 participants (Gender: 58% women, 42% men; Race: 70% White, 13% Black, 8% Asian, 8% biracial/other; Age: *M* = 38.9 years, *SD* = 14.7 years, min = 20, max = 77).

#### Stimuli and procedure

The ensemble stimuli were the same as in Study 1. Participants completed a consent form before verifying that they were wearing headphones in a quiet environment. Participants were exposed to 3 different counterbalanced blocks. Two of these blocks were identical to Study 1 and collected estimates of sex ratio and average face gender. The third block randomly presented 50 ensembles to participants and asked them how threatening they perceived the group to be on a scale from 1 (*not at all threatening*) to 7 (*extremely threatening*). Lastly, participants provided demographic information and were debriefed.

### Results

As expected, we replicated the accuracy results found in Study 1. As the ratio of men to women increased, so did participants’ numeric estimates of men in an ensemble, *B* = 0.84, *SE* = 0.04, *t*(105) = 23.03, *p* < 0.001. We also replicated the average group member appearance findings from Studies 1a and b. As the number of men in the group increased, participants perceived the average group member to be more masculine, *B* = 0.85, *SE* = 0.03, *t*(86) = 32.50, *p* < 0.001.

Importantly, we investigated whether the sex ratio of the group affected perceptions of threat. Regressing the overall threat judgment score onto the actual sex ratio, we found that as the ratio of men to women increased, threat judgments also increased, *B* = 0.11, *SE* = 0.02, *t*(81) = 6.68, *p* < 0.001. As expected, these results suggest that threat judgments are sensitive to group sex composition.

### Discussion

The general findings from Study 1 were replicated in Study 2 (Table 1). Listeners were accurate at judging the sex ratio of a group from their voices alone, and groups with more men were estimated to appear more facially masculine on average. Importantly, the sex composition of a group had implications for perceived threat. As the number of men in the group increased, so did threat judgments.

## Study 3

The goal of Study 3 was to investigate whether group sex composition influenced social belonging. Participants listened to ensembles and estimated the sex ratio of the group, the perceived average facial appearance of the group, and their perception of fit within the group. Because feelings of belonging in a group are related to the number of people who hold similar identities to you^26^, we predicted that reported belonging would increase the number of same-gender others increased.

### Method

#### Participants

84 participants from Prolific completed this 30-min study in exchange for $4.50. Three participants were excluded from analyses due to identical responses on each response item. One participant was excluded because they did not report demographic information and we investigated participant gender as a potential moderator. This yielded a final sample of 80 participants (Gender: 59% women, 41% men; Race: 75% White, 8% Black, 6% Asian, 11% biracial/other; Age: *M* = 38.9 years, *SD* = 12.8 years, min = 18, max = 73).

#### Stimuli

The ensemble stimuli were created in the same way as Studies 1 and 2.

#### Procedure

Participants completed a consent form and verified that they were wearing headphones in a quiet environment. Like Study 2, they were randomly presented 3 counterbalanced blocks. Each block played 50 ensembles in randomized order, with 10 ensembles of each sex ratio. One block asked participants about the perceived sex ratio of the ensemble. Another block asked participants about the perceived average group member. The third block asked participants how much they felt they would fit or belong in the group on a scale from 1 (*not at all*) to 7 (*extremely*). Lastly, participants provided demographic information and were debriefed.

### Results

We replicated the general accuracy results found in Studies 1 and 2. As the ratio of men to women in a group increased, so did participants’ numeric estimates of men in an ensemble, *B* = 0.80, *SE* = 0.04, *t*(111) = 19.93, *p* < 0.001. Furthermore, as the ratio of men to women increased, participants perceived the average group member to be more masculine, *B* = 0.88, *SE* = 0.03, *t*(95) = 33.80, *p* < 0.001.

**Figure 3:**
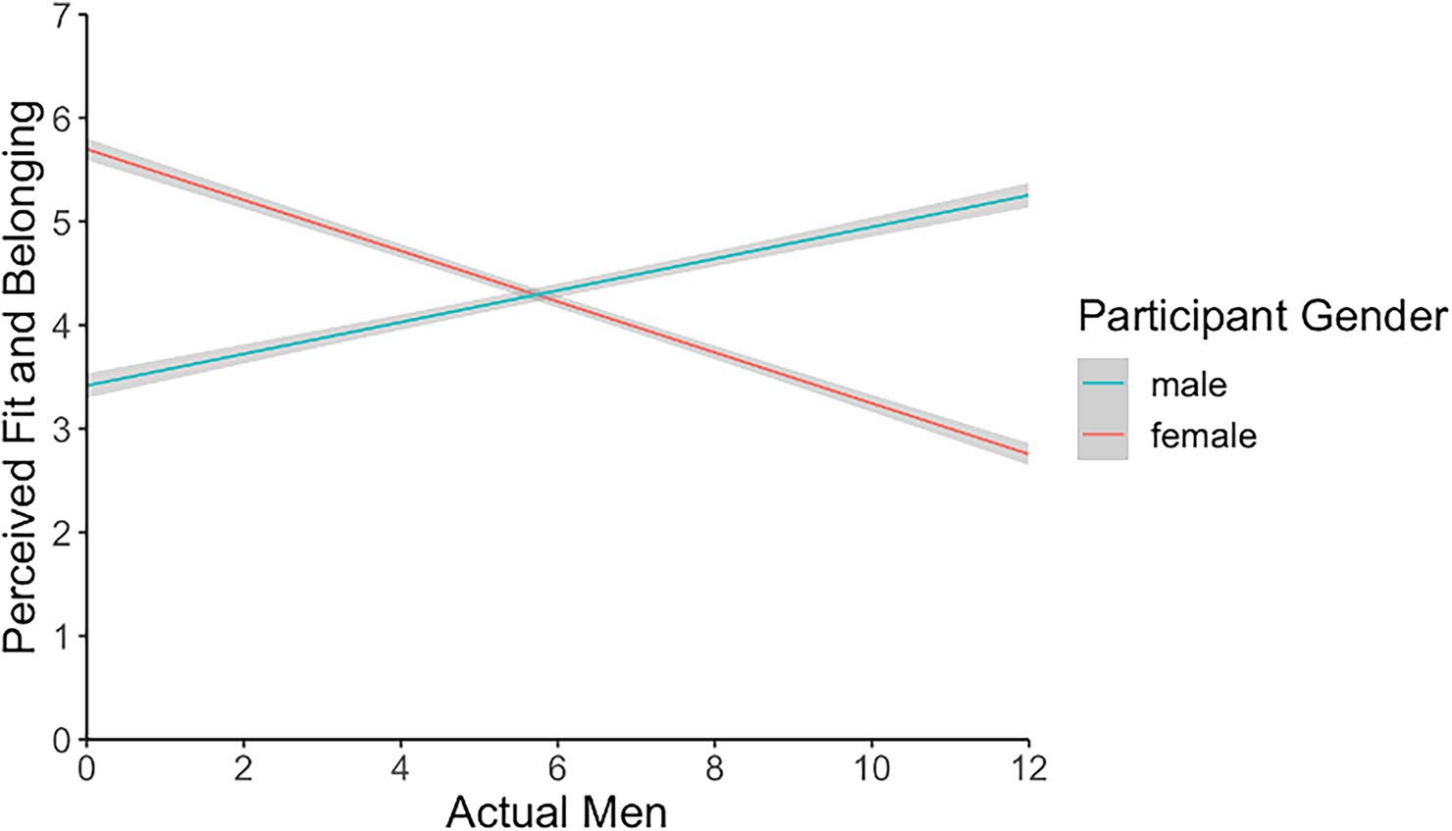
Study 3 perceived fit and belonging by actual sex ratio and participant gender. Note: Error bars represent 95% confidence intervals.

Next, we regressed perceived fit and belonging onto actual sex ratio. Perceived fit and belonging decreased as the group's ratio of men to women increased, *B* = − 0.08, *SE* = 0.03, *t*(87) = − 2.68, *p* = 0.009. Importantly, this trend was different depending on participant gender, *B* = 0.40, *SE* = 0.04, *t*(77) = 10.02, *p* < 0.001. Tests of simple slopes revealed that men felt that they belonged more when the ratio of men to women increased, *B* = 0.15, *SE* = 0.03, *t*(85) = 4.90, *p* < 0.001, whereas women felt that they fit less, *B* = − 0.25, *SE* = 0.03, *t*(88) = − 9.26, *p* < 0.001. We also tested how men and women perceivers’ feelings of fit and belonging varied when the actual number of men in the group was centered at three (a minority), six (equal representation), and nine (a majority). When groups consisted of three men and nine women, women perceivers (*M* = 4.91, *SD* = 1.32) reported significantly higher feelings of fit and belonging than men perceivers (*M* = 4.16, *SD* = 1.29), *B* = − 0.74, *SE* = 0.19, *t*(77) = − 3.83, *p* < 0.001. In groups with equal numbers of men and women, there was no significant between women perceivers (*M* = 4.40, *SD* = 1.45) and men perceivers (*M* = 4.57, *SD* = 1.16), *B* = 0.17, *SE* = 0.21, *t*(78) = 0.83, *p* = 0.409. In groups with nine men and three women, women perceivers (*M* = 3.84, *SD* = 1.55) felt significantly less belonging than men (*M* = 4.76, *SD* = 1.26), *B* = 2.67, *SE* = 0.32, *t*(78) = 8.43, *p* < 0.001 (Fig. 3).

### Discussion

The findings from Study 3 supported our hypothesis that as the number of men in a group increased, feelings of fit and belonging decreased. However, as we predicted, this trend differed significantly by participant gender. Men and women reported more belonging in groups with more same-gender members. The accuracy findings from the previous studies were replicated (Table 1).

## General discussion

Based on previous research on visual ensembles, we expected that listeners would be able to accurately judge the sex composition of auditory ensembles and that these categorizations would affect social judgments of groups. In particular, we expected that increased ratios of men in a group would be associated with perceptions of threat, and that feelings of belonging would be linked to perceivers’ gender, such that men would feel more belonging in groups with more men, and women would feel more belonging in groups with more women. All of these predictions were confirmed across our four studies. Listeners accurately judged the sex composition of a group from voices alone and used perceptual features to form mental representations of the average group member that embodied gendered features. Effect sizes for accuracy across studies are reported in Table 1 for comparison. Further, we found that the sex composition of a group had downstream consequences for evaluative inferences. As the number of men in the group increased, so did perceived threat (Study 2). This suggests that the ratio of men to women in a group affects judgments in a manner that is consistent with social stereotypes that characterize men as threatening. Meanwhile, perceived social belonging increased with the number of same-gender others in the group (Study 3).

These findings expand knowledge in multiple domains including ensemble coding, voice perception, interpersonal communication, and social cognition. Ensemble coding is largely focused on narrow aspects of visual perception but the mechanisms underlying ensemble coding extend to social stimuli as well. Our research complements and extends these findings to show that ensemble coding operates similarly in the auditory modality. Furthermore, we extend findings by Neuhoff and colleagues^40,41^ by demonstrating downstream consequences of extracting sex information from a vocal ensemble. Social groups generate a variety of auditory signals and cues that reveal aspects of their composition, intent, and experience^63^. We should expect a fine-tuned ability to extract sex composition from both the visual and auditory modalities in the service of important social judgments such as how much a group might either constitute a threat or possible alliance. These are the first studies to test the downstream consequences of rapidly extracting summary statistical information from vocal ensembles. Here, we show that people can make social judgments of threat and alliance rather effortlessly, relaying on accurate information gleaned from just brief exposures to auditory information alone. This suggests that social inferences of voices are shaped by a combination of low-level (e.g., pitch) and high-level (e.g., stereotype) information.

Furthermore, these findings have important implications for understanding why groups with historically unbalanced gender representation have resisted change. Our studies suggest that regardless of the specific context, women exposed to auditory cues indicating male-dominated groups may experience a diminished sense of fit or belonging. This judgment might subsequently influences one’s approach or avoidance behavior toward the group. Groups that are historically dominated by men (e.g., STEM fields) might deter women from applying due to lack of perceived fit and increased threat. This phenomenon can also occur for men, who might feel decreased fit despite perceived threat being relatively low in fields that are historically dominated by women such as nursing, education, or psychology^64,65^. Increasing gender representation across fields may positively influence equal participation of men and women.

### Future directions

The experimental methods used in this paper allow us to draw strong conclusions about the causal mechanisms underlying threat and belonging judgments by using artificially created ensembles. Future research should examine if these findings hold in real-world vocal ensembles or in ensembles with overlapping or disorganized voices that simulate various group vocalization scenarios. For example, investigating how other social identities conveyed through the voice (e.g., accent, age) influence judgments of threat and belonging would be informative. To enhance ecological validity, future studies could also manipulate semantic content reflective of various environments (e.g., STEM) and collect behavioral measures along with self-reported measures of threat and belonging.

The findings reported here provide a strong foundation for testing the relative contributions of low- and high-level information on evaluative judgments that parallel existing research in the visual literature^66^. That is, research could systematically test whether judgments are affected more strongly by low-level perceptual features of the voice (e.g., pitch and timbre) or by the high-level stereotypes held toward the categories of men and women by manipulating the relative pitch within men and women’s voices to be typical (high-pitched women, low-pitched men) or atypical (low-pitched women, high-pitched men). If judgments are affected only by category information communicated through the voice, then perceived threat and fit should only vary as a function of the sex ratio of the group. However, if judgments are also sensitive to within-sex perceptual variations in the voice, then perceived threat should be exaggerated in groups with more masculinized voices and attenuated in groups with more feminized voices, regardless of actual sex.

It is important to note that our studies build on binary assumptions of sex and gender, and thus future research should test (i) if perceivers discern group composition beyond this binary framework, and (ii) how nonbinary or genderqueer individuals evaluate groups with varying sex ratios. Social cognitive research demonstrates that people automatically categorize others into binary sex and gender groups with minimal control, and form impressions based on activated category stereotypes^67,68^. However, as societal recognition of nonbinary and genderqueer identities grows, mental representations may expand beyond the binary. This could lead to more nuanced numerosity judgments of group composition beyond males and females or men and women. Additionally, while our findings demonstrate that men and women feel a stronger sense of belonging in groups with similar others, we could not assess this for nonbinary or genderqueer participants due to insufficient power. Future research should explore this area to enhance our understanding of the interplay of identity and group perception.

In most communication environments, perceivers receive both visual and auditory information simultaneously. However, very little work has investigated how information from multiple sensory domains combine to influence social categorizations and judgments. Although some work exists looking at multimodal perception of individuals^69–74^, extending this research to group settings would contribute to more ecologically valid theories of ensemble perception.

### Conclusion

We expected perceptual sensitivity to auditory information in assessing groups given that visual information is often not available in intergroup interactions. In four studies, we demonstrated that individuals are highly attuned to the sex ratios of groups after briefly hearing their voices, and that information about group composition can be extracted efficiently and implicitly. Furthermore, the composition of groups influenced perceptions of group threat and belonging such that threat increased concomitantly with the number of men in the group and belonging was tethered to the number of same-gender others in the group.

## Data Availability

The data, code, and materials that support the findings of this paper are openly available on OSF (https://osf.io/28gvd/).
